# Draft genome sequence of *Methylibium* sp. strain T29, a novel fuel oxygenate-degrading bacterial isolate from Hungary

**DOI:** 10.1186/s40793-015-0023-z

**Published:** 2015-07-19

**Authors:** Zsolt Szabó, Péter Gyula, Hermina Robotka, Emese Bató, Bence Gálik, Péter Pach, Péter Pekker, Ildikó Papp, Zoltán Bihari

**Affiliations:** Bay Zoltán Nonprofit Ltd. for Applied Research, Budapest, Hungary; Materials Science Research Group, Hungarian Academy of Sciences-University of Miskolc, Miskolc, Hungary

**Keywords:** *Methylibium*, *Betaproteobacteria*, Draft genome, Fuel oxygenates, Bioremediation

## Abstract

*Methylibium* sp. strain T29 was isolated from a gasoline-contaminated aquifer and proved to have excellent capabilities in degrading some common fuel oxygenates like methyl *tert*-butyl ether, *tert*-amyl methyl ether and *tert*-butyl alcohol along with other organic compounds. Here, we report the draft genome sequence of *M.* sp. strain T29 together with the description of the genome properties and its annotation. The draft genome consists of 608 contigs with a total size of 4,449,424 bp and an average coverage of 150×. The genome exhibits an average G + C content of 68.7 %, and contains 4754 protein coding and 52 RNA genes, including 48 tRNA genes. 71 % of the protein coding genes could be assigned to COG (Clusters of Orthologous Groups) categories. A formerly unknown circular plasmid designated as pT29A was isolated and sequenced separately and found to be 86,856 bp long.

## Introduction

Fuel oxygenates like MTBE, ETBE and TAME have been blended into gasoline for decades to boost octane ratings and to improve the efficiency of fuel combustion in engines. But being the most water-soluble components of gasoline they have simultaneously become some of the most frequently detected pollutants in groundwater posing a serious threat to drinking water supplies [1]. Moreover, recent studies have reported that they can be carcinogenic in humans [2], so remediation of the sites polluted with these compounds became an important issue. Several microbial consortia and individual bacterial strains were isolated so far being capable of their degradation to various extents [3, 4]. However, only a few of them were studied in detail and there are even fewer cases where the genetic and enzymatic background of the degradation is elucidated at least in some aspects.

*Methylibium petroleiphilum* PM1 was one of the first isolated individual MTBE-degrading strains originated from a compost-filled biofilter in Los Angeles, California, USA [5]. To date it is the only representative of the genus identified at the species level [6, 7]. During laboratory experiments it proved to have outstanding MTBE-degrading ability and it was tested in a bioaugmentation field study, too [8]. Afterwards, a number of bacteria closely related to *M. petroleiphilum* PM1 were detected based on 16S rDNA sequences at MTBE-contaminated sites at different geographic locations suggesting that the genus might have an important role in MTBE biodegradation [8, 9]. Later its complete genome sequence was published which revealed that besides the 4 Mb circular chromosome, *M. petroleiphilum* PM1 possesses a ~600 kb megaplasmid carrying the genes involved in MTBE degradation [10]. At present, no genome sequence information is available for other members of the *Methylibium* genus. As part of a French-Hungarian project aiming to characterize novel fuel oxygenate-degrading bacteria at the genomic level, we have isolated a novel *Methylibium* strain. The MTBE-degrading capacity of the strain was as high as the *M. petroleiphilum* PM1’s but some of its genetic and metabolic characteristics were found to be significantly different. Here we present the classification and features of *Methylibium* sp. T29 together with the description of the draft genome sequence and annotation compared to the reference strain *M. petroleiphilum* PM1.

## Organism information

### Classification and features

**Fig. 1:**
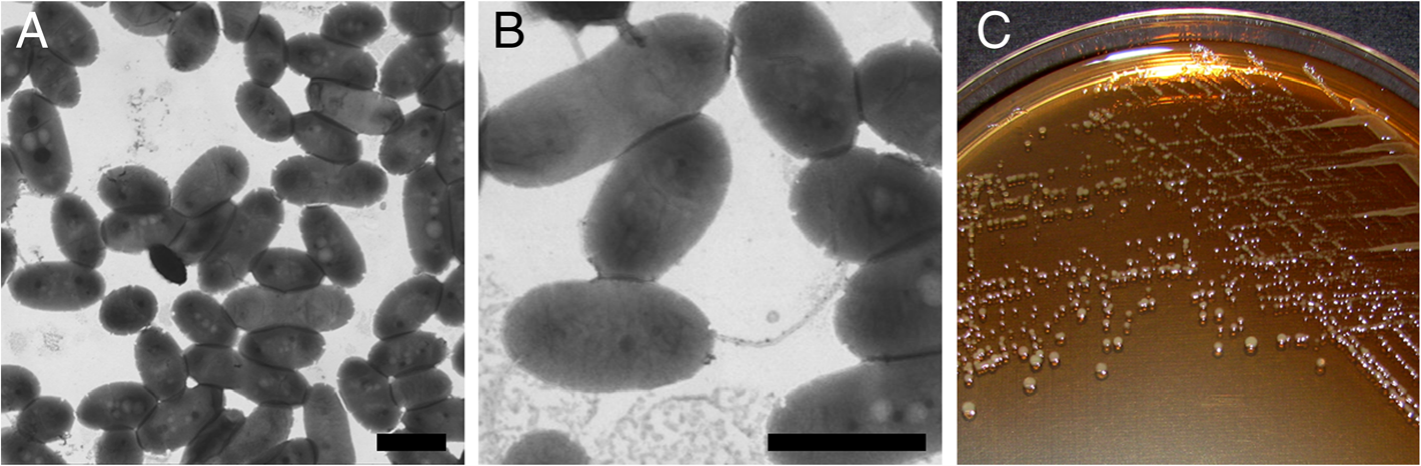
Transmission electron micrographs (**a** and **b**) and extracellular pigment production (**c**) of *Methylibium* sp. T29. For TEM examination the cells were suspended in 18 MΩ ultra-pure water, and 10 μl of the cell suspension was placed on carbon- and Formvar-coated 300 Mesh copper grids. Single 10 μl drops of 1 % (w/v) aqueous uranyl acetate were added to the grid for 15 s. The images were taken on a Hitachi S-4800 type (FEG) scanning electron microscope in transmission mode using 25 kV acceleration voltage. Scale bars represent 1 μm. The morphology of the cells is similar to *M. petroleiphilum* PM1’s [6]. While grown on ½ × TSA plates *M.* sp. T29 secreted a brownish pigment resembling pyomelanin produced by certain *Pseudomonas* spp

A novel potent MTBE-degrading bacterial strain designated as T29 was isolated from a mixed bacterial culture enriched from gasoline-contaminated groundwater samples collected from the area of Tiszaújváros, Hungary. The enrichment culture was supplemented with *tert*-butyl alcohol (TBA), one of the known key intermediates of MTBE biodegradation, as the sole carbon source. The strain was found to be able to utilize the following compounds provided as the sole carbon and energy sources: MTBE, TAME, TBA, 2-HIBA, benzene, methanol, ethanol, 1-propanol, 1-butanol, formate, piruvate and acetate, but cannot grow on ETBE, DIPE, *n*-alkanes, toluene, ethylbenzene, *o*-, *m*- and *p*-xylene, 2-propanol, acetone, formaldehyde, lactate, citrate and glucose. Strain T29 was routinely maintained in mineral salts medium (124 mg/l (NH_4_)_2_SO_4_, 50 mg/l MgSO_4_ · 7H_2_O, 12.5 mg/l CaCl_2_ · 2H_2_O, 350 mg/l KH_2_PO_4_, 425 mg/l K_2_HPO_4_, 1 mg/l FeSO_4_ · 7H_2_O, 1 mg/l CoCl_2_ · 6H_2_O, 1 mg/l MnSO_4_ · H_2_O, 1 mg/l ZnSO_4_ · 7H_2_O, 1 mg/l Na_2_MoO_4_ · 2H_2_O, 1 mg/l Na_2_WO_4_ · 2H_2_O, 0.25 mg/l NiCl_2_ · 6H_2_O, 0.1 mg/l H_3_BO_3_, 0.1 mg/l CuSO_4_ · 5H_2_O and 1.5 % agar if necessary) containing 200 mg/l MTBE or in ½ × TSB medium (8.5 g/l pancreatic digest of casein, 1.5 g/l papaic digest of soybean meal, 2.5 g/l NaCl, 1.25 g/l K_2_HPO_4_, 1.25 g/l glucose and 1.5 % agar if necessary) at 28 °C. Cells of strain T29 form pale yellow, shiny colonies on minimal agar plates and cream colored ones on ½ × TSA plates while secreting a brownish pigment molecule (Fig. 1, panel c) reminiscent of pyomelanin produced by certain *Pseudomonas* spp. and other strains belonging mainly to *Gammaproteobacteria* [11, 12]. Strain T29 stained Gram-negative and according to transmission electron micrographs (Fig. 1, panel a and b) the cell shape is coccobacillus. A smaller fraction of the cell population possesses a single polar flagellum (Fig. 1, panel b). Possible intracellular poly-β-hydroxyalkanoate granules (white spots) and possible protein inclusion bodies (dark spots) can also be observed.

**Fig. 2:**
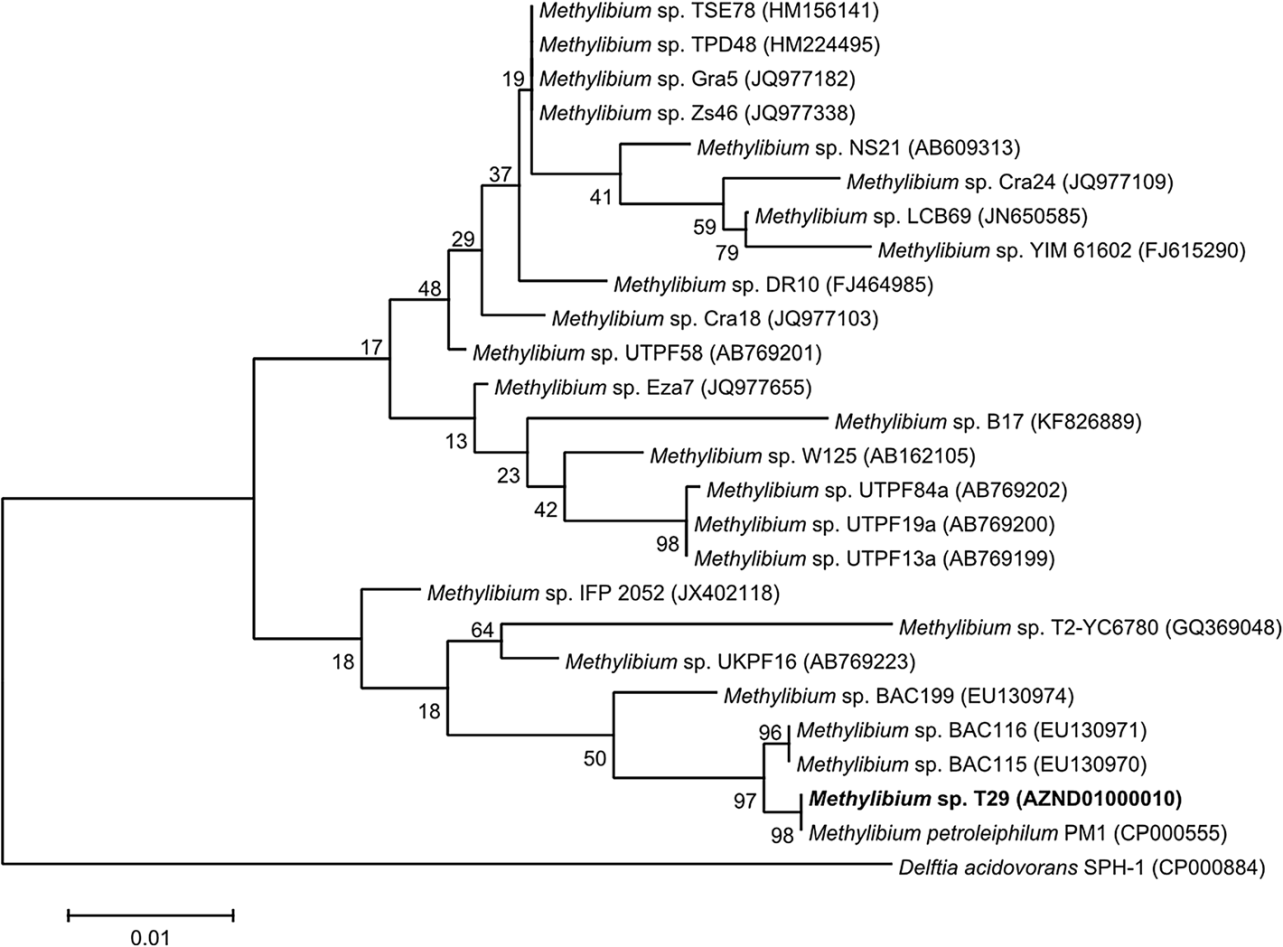
Dendrogram indicating the phylogenetic relationships of *Methylibium* sp. T29 relative to other *Methylibium* isolates. The maximum likelihood tree was inferred from 1329 aligned positions of the 16S rRNA gene sequences and derived based on the Tamura-Nei model using MEGA 6 [15]. *Delftia acidovorans* SPH-1 was used as an outlier. Bootstrap values (expressed as percentages of 1000 replicates) are shown at branch points. Bar: 0.01 substitutions per nucleotide position. The corresponding GenBank accession numbers are displayed in parentheses

Initial taxonomic assignment of the strain was established by comparing its 16S ribosomal RNA gene sequence to the nonredundant Silva SSU Ref database [13, 14]. Phylogenetic analysis was conducted using MEGA 6 [15]. According to the phylogenetic analysis, strain T29 belongs to the genus *Methylibium* (Table 1). The closest relative of strain T29 is *M. petroleiphilum* PM1 (Fig. 2).

**Table 1:** Classification and general features of Methylibium sp. strain T29 according to the MIGS recommendation [37]

MIGS ID	Property	Term	Evidence code^a^
	Classification	Domain *Bacteria*	TAS [38]
		Phylum *Proteobacteria*	TAS [39]
		Class *Betaproteobacteria*	TAS [40, 41]
		Order *Burkholderiales*	TAS [41, 42]
		Family *Comamonadaceae*	TAS [43, 44]
		Genus *Methylibium*	TAS [6, 7]
		Species *Methylibium* sp.	IDA
		Strain T29	IDA
	Gram stain	Negative	IDA
	Cell shape	Coccobacillus	IDA
	Motility	Motile	IDA
	Sporulation	Not reported	NAS
	Temperature range	Mesophilic	IDA
	Optimum temperature	28 °C	IDA
	pH range; Optimum	Not determined; routinely grown at pH 6.5	IDA
	Carbon source	MTBE; TAME; TBA; methanol; ethanol	IDA
MIGS-6	Habitat	Soil; Groundwater	IDA
MIGS-6.3	Salinity	Not reported	NAS
MIGS-22	Oxygen requirement	Aerobic	IDA
MIGS-15	Biotic relationship	Free living	NAS
MIGS-14	Pathogenicity	Non-pathogenic	NAS
MIGS-4	Geographic location	Tiszaújváros, Hungary	IDA
MIGS-5	Sample collection	Nov-2010	IDA
MIGS-4.1	Latitude	47.9179167	IDA
MIGS-4.2	Longitude	21.0285667	IDA
MIGS-4.4	Altitude	94 m	IDA

^a^Evidence codes – IDA: Inferred from Direct Assay; TAS: Traceable Author Statement (i.e., a direct report exists in the literature); NAS: Non-traceable Author Statement (i.e., not directly observed for the living, isolated sample, but based on a generally accepted property for the species, or anecdotal evidence). These evidence codes are from the Gene Ontology project [45]

**Table 2:** Genome sequencing project information

MIGS ID	Property	Term
MIGS-31	Finishing quality	Draft
MIGS-28	Libraries used	One 200 bp Ion Torrent library
MIGS-29	Sequencing platforms	Ion Torrent PGM
MIGS-31.2	Fold coverage	150×
MIGS-30	Assemblers	GS De Novo Assembler 2.9
MIGS-32	Gene calling method	Prodigal 2.6, Barrnap 0.3, Aragorn 1.2 (as part of Prokka 1.8)
	Locus Tag	X551
	Genbank ID	AZND00000000
	Genbank Date of Release	2014/02/20
	GOLD ID	Gp0074688
	BIOPROJECT	PRJNA229978
MIGS-13	Source Material Identifier	SAMN02422539
	Project relevance	Environmental, biotechnology

Despite its close relatedness based on 16S rDNA sequences, the new strain differs from the type strain *M. petroleiphilum* PM1 in several aspects. For example, unlike *M. petroleiphilum* PM1, strain T29 is resistant to tetracycline, ampicillin [16] and mercury, and cannot grow on *n*-alkanes [10]. Moreover, PCR primers designed for *mdpA* and other known genes involved in MTBE degradation in *M. petroleiphilum* PM1 [17] failed to detect any related sequences in strain T29 suggesting that the genetic makeup of MTBE metabolism in this strain differs significantly from the one in *M. petroleiphilum* PM1. Pulsed field gel electrophoresis of restriction enzyme digested genomic DNA of strain T29 and *M. petroleiphilum* PM1 revealed major differences in the genomic sequences of the two strains (data not shown). Based on the evidences above, the new strain was named as *Methylibium* sp. T29.

## Genome sequencing information

### Genome project history

The genome of *M*. sp. T29 was sequenced by using Ion Torrent technology in our facility. The draft genome was assembled *de novo* using the overlap layout consensus methodology by the freely available software GS De Novo Assembler 2.9 (Roche). This Whole Genome Shotgun project has been deposited at DDBJ/EMBL/GenBank under the accession number AZND00000000. The version described in this paper is AZND01000000. The plasmid pT29A was isolated and sequenced separately by the same technology. The assembly was performed by a different approach using SPAdes 3.0 [18]. The sequence was circularized and finished by manual editing. The full sequence of the plasmid pT29A is also available in GenBank under the accession number NC_024957.1.

### Growth conditions and genomic DNA preparation

*M*. sp. T29 was isolated from a mixed bacterial culture enriched from gasoline-contaminated groundwater samples collected from the area of Tiszaújváros, Hungary, in November 2010. The strain was deposited into the National Collection of Agricultural and Industrial Microorganisms (NCAIM) [19] under the accession number NCAIM B.02561.

For genomic DNA preparation, bacteria were grown under aerobic conditions in a tightly sealed bottle at 28 °C for 14 days in mineral salts medium supplemented with 200 mg/l MTBE. Genomic DNA was isolated using UltraClean Microbial DNA Isolation Kit (MO BIO) according to the protocol provided by the manufacturer.

### Genome sequencing and assembly

The genomic library was prepared using IonXpress Plus Fragment Library Kit (Life Technologies) and was sequenced using Ion PGM 200 Sequencing Kit v2 with an Ion Torrent PGM Sequencer. The raw data were processed using Torrent Suite 4.0.1. The number of usable reads was 3,100,682 with a total base number of 690,903,502. The mean read length was 222.82 ± 41.88 bp, the mode length was 243 bp. Contigs were built *de novo* using GS De Novo Assembler 2.9 (Roche). The assembly resulted in 608 contigs, the largest contig size was 98,303 bp, the minimum contig size was 505 bp. The half of the genome consists of contigs larger than 15,441 bp (N50). The average coverage was 150 × (Table 2).

The pT29A plasmid was purified using a modified plasmid miniprep method [20] and treated with Plasmid-Safe™ ATP-dependent DNase (Epicentre) before sequencing with Ion Torrent technology using the kits mentioned above. 40,770 reads were obtained with a total base number of 8,500,697. The mean read length was 208.50 ± 51.50 bp, the mode length was 234 bp. The reads were assembled into an 86,856 bp circular sequence with SPAdes 3.0 [18] and manual editing.

### Genome annotation

The assembled draft genome and the pT29A sequences were annotated using Prokka 1.8 [21]. For the prediction of signal peptides and transmembrane domains SignalP 4.1 Server [22, 23] and TMHMM Server v. 2.0 [24] were used, respectively. Assignment of genes to the COG database [25, 26] and Pfam domains [27] was performed with WebMGA server [28].

## Genome properties

**Fig. 3:**
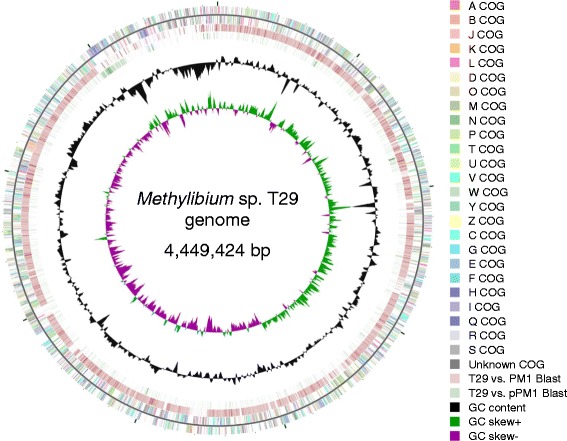
Circular representation of the draft genome of *Methylibium* sp. T29 displaying relevant genome features. The contigs of *M.* sp. T29 were reordered by Mauve [35] using the genome sequence of *M. petroleiphilum* PM1 as the reference. The COG categories were assigned to genes by WebMGA [28]. The circular map was visualized by CGView [36]. The features are the following from outside to center: (A) genes on forward strand; genes on reverse strand (colored by COG categories); blast alignment of the *M. petroleiphilum* PM1 chromosome and megaplasmid to the draft genome of *M.* sp. T29; GC content; GC skew

**Fig. 4:**
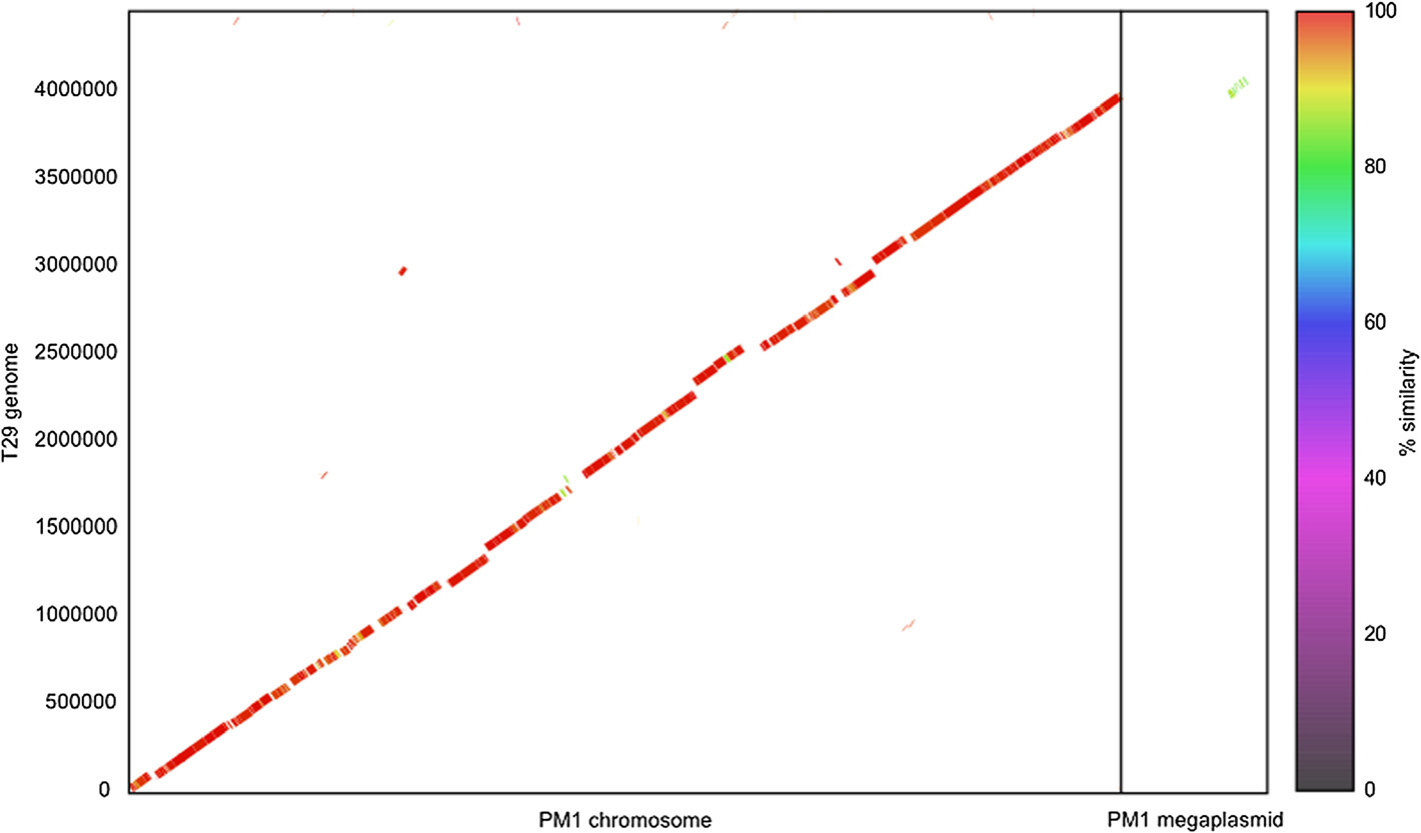
Genome sequence similarity plot of *Methylibium* sp. T29 and *Methylibium petroleiphilum* PM1. Contigs from the draft genome assembly of *M.* sp. T29 were reordered with Mauve 2.3.1 [35] using the complete genome of *M. petroleiphilum* PM1 as the reference. The alignment and plotting were performed with MUMmer 3.0 [29]

**Fig. 5:**
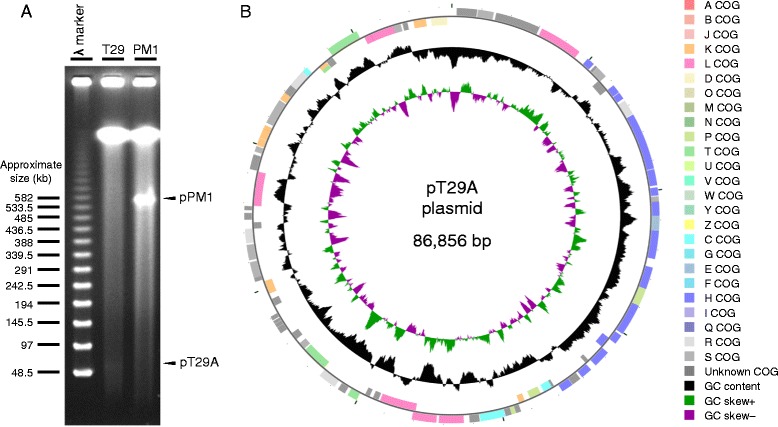
Detection and features of the pT29A plasmid. **a** Separation of megaplasmids of *M. petroleiphilum* PM1 and *M.* sp. T29 by pulsed field gel electrophoresis. The experiment was conducted according to Barton *et al*. [30]. The arrows show the ~600 kb partially linearized megaplasmid of *M. petroleiphilum* PM1 described in [10], and the ~87 kb partially linearized pT29A plasmid described in this paper. **b** Circular representation of the pT29A plasmid of *M.* sp. T29 displaying relevant features. The circular map was visualized by CGView [36]. The features are the following from outside to center: genes on forward strand, genes on reverse strand (colored by COG categories), GC content and GC skew

The total size of the draft genome of *M*. sp. T29 is 4,449,424 bp and has a G + C content of 68.7 % which is similar to the genome of the type strain *M. petroleiphilum* PM1 (4,643,669 bp, G + C content of 67.6 %). For *M*. sp. T29 a total of 4806 genes, whilst for *M. petroleiphilum* PM1 4477 genes were predicted. 3 rRNA, 48 tRNA and 1 tmRNA genes were detected in the genome of *M.* sp. T29. We could make functional prediction for 72.8 % of the protein coding genes, while the rest were named as hypothetical proteins. Of the coding genes, 71 % could be assigned to COG categories and 71.4 % has Pfam domains (for detailed statistics see Tables 3 and 4). The map of the draft genome of *M.* sp. T29 aligned to the full genome of the closest relative *M. petroleiphilum* PM1 is illustrated in Fig. 3 and Fig. 4. The plasmid pT29A carries 90 protein coding genes, of which 72.2 % has functional prediction and 70 % could be assigned to COG categories (Table 5). The most abundant functional category was the coenzyme transport and metabolism (Table 6). The map of the plasmid is shown in Fig. 5.

**Table 3:** Genome statistics

Attribute	Value	%age of total
Genome size (bp)	4,449,424	100
DNA coding (bp)	3,743,112	84.1
DNA G + C (bp)	3,057,506	68.7
DNA scaffolds	608	n/a
Total genes	4806	n/a
Protein coding genes	4754	98.9
RNA genes	52	1.1
Pseudo genes	196	4.1
Genes in internal clusters	N.D.	N.D.
Genes with function prediction	3498	72.8
Genes assigned to COGs	3376	71.0
Genes with Pfam domains	3395	71.4
Genes with signal peptides	381	8.0
Genes with transmembrane helices	1014	21.3
CRISPR repeats	0	0

**Table 4:** Number of genes associated with general COG functional categories in the whole genome

Code	Value	%age	Description
J	169	3.5	Translation, ribosomal structure and biogenesis
A	2	0.0	RNA processing and modification
K	276	5.8	Transcription
L	190	4.0	Replication, recombination and repair
B	4	0.1	Chromatin structure and dynamics
D	32	0.7	Cell cycle control, Cell division, chromosome partitioning
V	59	1.2	Defense mechanisms
T	284	6.0	Signal transduction mechanisms
M	218	4.6	Cell wall/membrane biogenesis
N	100	2.1	Cell motility
U	122	2.6	Intracellular trafficking and secretion
O	170	3.6	Posttranslational modification, protein turnover, chaperones
C	292	6.1	Energy production and conversion
G	126	2.6	Carbohydrate transport and metabolism
E	295	6.2	Amino acid transport and metabolism
F	72	1.5	Nucleotide transport and metabolism
H	196	4.1	Coenzyme transport and metabolism
I	177	3.7	Lipid transport and metabolism
P	236	5.0	Inorganic ion transport and metabolism
Q	118	2.5	Secondary metabolites biosynthesis, transport and catabolism
R	456	9.6	General function prediction only
S	337	7.1	Function unknown
-	823	17.3	Not in COGs

The total is based on the total number of protein coding genes in the genome

**Table 5:** Statistics for the pT29A plasmid

Attribute	Value	%age of total
Genome size (bp)	86,856	n.a.
DNA coding (bp)	75,837	87.3
DNA G + C (bp)	58,265	67.1
DNA scaffolds	1	100.0
Total genes	90	100.0
Protein coding genes	90	100.0
RNA genes	0	0.0
Pseudo genes	1	1.1
Genes in internal clusters	N.D.	N.D.
Genes with function prediction	65	72.2
Genes assigned to COGs	63	70.0
Genes with Pfam domains	67	74.4
Genes with signal peptides	12	13.3
Genes with transmembrane helices	17	18.9
CRISPR repeats	0	0.0

**Table 6:** Number of genes associated with general COG functional categories in the pT29A plasmid genome

Code	Value	%age	Description
J	0	0.0	Translation, ribosomal structure and biogenesis
A	0	0.0	RNA processing and modification
K	8	8.9	Transcription
L	10	11.1	Replication, recombination and repair
B	4	0.1	Chromatin structure and dynamics
D	1	1.1	Cell cycle control, Cell division, chromosome partitioning
V	0	0.0	Defense mechanisms
T	7	7.8	Signal transduction mechanisms
M	0	0.0	Cell wall/membrane biogenesis
N	0	0.0	Cell motility
U	0	0.0	Intracellular trafficking and secretion
O	0	0.0	Posttranslational modification, protein turnover, chaperones
C	3	3.3	Energy production and conversion
G	0	0.0	Carbohydrate transport and metabolism
E	1	1.1	Amino acid transport and metabolism
F	0	0.0	Nucleotide transport and metabolism
H	19	21.1	Coenzyme transport and metabolism
I	0	0.0	Lipid transport and metabolism
P	5	5.6	Inorganic ion transport and metabolism
Q	0	0.0	Secondary metabolites biosynthesis, transport and catabolism
R	4	4.4	General function prediction only
S	10	11.1	Function unknown
-	22	24.4	Not in COGs

The total is based on the total number of protein coding genes in the plasmid genome

## Conclusions

On average, the draft genome of *M.* sp. T29 shows 97 % identity to the *M. petroleiphilum* PM1 chromosome and 85 % identity to a small part of the *M. petroleiphilum* PM1 megaplasmid at the nucleotide level as measured by NUCmer [29] (Fig. 4) but significant differences were also found. Notably, most parts of the 600 kb megaplasmid are missing from *M.* sp. T29. A pulsed field gel electrophoretic analysis to detect megaplasmids [30] revealed that unlike *M. petroleiphilum* PM1 our isolate does not harbor the megaplasmid which carries the genes for MTBE-degradation [10]. Instead, a ~87 kb plasmid is present (Fig. 5) that we named pT29A.

The fact that in *M. petroleiphilum* PM1 the genes for MTBE-metabolism are located on the pPM1 megaplasmid suggested that in *M.* sp. T29 these genes are also carried by the pT29A plasmid. Surprisingly, no known genes associated with MTBE-degradation were found among the plasmid coded genes besides a cobalamin-synthesis operon which differs from the one in *M. petroleiphilum* PM1. Cobalt ions or cobalamin are required for complete MTBE-degradation in some strains for the utilization of 2-HIBA which is a key intermediate in the metabolic pathway [31, 32]. However, we were able to identify the putative components of the MTBE-degradation pathway in the whole genome of the *M.* sp. T29 including orthologous genes coding for the MTBE monooxygenase [16] and the TBA monooxygenase [33] showing only 84 and 81 % identity at the amino acid level to their *M. petroleiphilum* PM1 counterparts, respectively (Table 7). As opposed to the considerably high similarity of the majority of the two genomes, the significantly lower sequence conservation of the MTBE-degradation pathway components and the fact that these genes are not linked to the pT29A plasmid indicate that the gene cluster for MTBE-metabolism is probably located on a transposon which resides on the megaplasmid and the chromosome in *M. petroleiphilum* PM1 and *M.* sp. T29, respectively. There are unique sequences in the *M.* sp. T29 genome missing from *M. petroleiphilum* PM1 conferring different functions, i.e. resistances to different antibiotics (ampicillin, meticillin, tetracycline, sulfonamide), heavy metals (mercury, copper, cobalt, nickel, zinc, cadmium, tellurium) and other toxic compounds (i.e. arsenic). Other unique sequences code for various metabolic enzymes, transcriptional regulators, sensor proteins, components of restriction modification systems, phage- and transposon-related proteins and hypothetical proteins. The MTBE monooxygenase function for the candidate gene *mdpA* and the resistances to ampicillin, tetracycline and mercury were verified experimentally. According to the gene annotations, *M.* sp. T29 can utilize other environmentally polluting compounds as well (i.e. chlorinated aromatic hydrocarbons, haloacids and certain polycyclic aromatic hydrocarbons) but these functions have not been tested yet. The organism was predicted as non-human pathogen (probability of being a human pathogen is 0.083) by PathogenFinder 1.1 [34], therefore it can be safely applied during *in situ* bioremediation experiments. Based on the genome sequence described here we designed PCR primers specific to the *M.* sp. T29-type *mdpA* to track our strain in the field at MTBE-contaminated sites in Hungary. The nucleotide sequences of other genes in the MTBE-degradation pathway can also be used to construct better oligonucleotide chips to detect the potentially active genes in environmental samples.

**Table 7:** Genes involved in the degradation of MTBE in Methylibium petroleiphilum PM1 and Methylibium sp. T29

Gene function	Gene ID in *M. petroleiphilum* PM1	Gene ID in *M.* sp. T29	%age identity at the nucleic acid level	%age identity at the amino acid level
MTBE monooxygenase	Mpe_B0606	X551_03232	79	84
Rubredoxin	Mpe_B0602	X551_03234	no significant similarity	43
Rubredoxin reductase	Mpe_B0597	X551_01331	no significant similarity	29
ATP-dependent transcriptional regulator	Mpe_B0601	X551_04638	74	85
Hydroxymethyl *tert*-butyl ether dehydrogenase	Mpe_B0558	X551_02800	86	91
*tert*-butyl formate carboxylesterase	Mpe_A2443	X551_01122	99	99
*tert*-butyl alcohol hydroxylase	Mpe_B0555	X551_02402	79	81
Iron-sulfur oxidoreductase	Mpe_B0554	X551_02401	82	82
2-methyl-2-hydroxy-1-propanol dehydrogenase	Mpe_B0561	X551_02804	83	85
Hydroxyisobutyraldehyde dehydrogenase	Mpe_A0361	X551_03863	Partial homology	36
2-hydroxy-isobutyryl-CoA ligase	Mpe_B0539	X551_02557	85	94
2-hydroxy-isobutyryl-CoA mutase	Mpe_B0541	X551_02559	89	92
2-hydroxy-isobutyryl-CoA mutase C-terminal domain	Mpe_B0538	X551_02556	86	91
3-hydroxybutyryl-CoA dehydrogenase	Mpe_B0547	X551_02564	79	84
Acetyl-CoA acetyltransferase	Mpe_A3367	X551_00431	Partial homology	45

## Abbreviations

MTBE: Methyl *tert*-butyl ether
ETBE: Ethyl *tert*-butyl ether
TAME: *Tert*-amyl methyl ether
TBA: *Tert*-butyl alcohol
2-HIBA: 2-hydroxyisobutyric acid
DIPE: Diisopropyl ether
TSA: Tryptic soy agar
TSB: Tryptic soy broth
